# Association between transcutaneous oxygen saturation within 24 h of admission and mortality in critically ill patients with non-traumatic subarachnoid hemorrhage: a retrospective analysis of the MIMIC-IV database

**DOI:** 10.3389/fneur.2023.1292260

**Published:** 2023-11-20

**Authors:** Junjie Liu, Zongxu Zhao, Jianmin Li, Qiuhua Zhang, Yichao Wang, Junwei Zhang

**Affiliations:** ^1^College of Clinical Medicine, North China University of Science and Technology, Tangshan, China; ^2^College of Stomatology, North China University of Science and Technology, Tangshan, China; ^3^Department of Neurosurgical Intensive Care Unit, The Affiliated Hospital, North China University of Science and Technology, Tangshan, China

**Keywords:** transcutaneous oxygen saturation, mortality, hypoxia, hyperoxia, subarachnoid hemorrhage, intensive care unit

## Abstract

**Background:**

In critically ill patients, transcutaneous oxygen saturation (SpO_2_) upon admission is typically associated with in-hospital mortality. Nevertheless, the available information for patients with non-traumatic subarachnoid hemorrhage (SAH) is limited. In our study, our objective was to assess the correlation between SpO_2_ levels and mortality among patients diagnosed with severe SAH.

**Methods:**

In this study, we extracted data from the Medical Information Marketplace in Intensive Care (MIMIC-IV) database, which comprises information on critically ill patients. By employing matching ICD-9 and ICD-10 codes, we identified 3,328 patients diagnosed with SAH. Every individual who was admitted to the intensive care unit (ICU) had their SpO_2_ data and various covariates, including age, sex, diagnosis, and duration of stay, recorded upon admission. Subsequently, the patients were categorized into three distinct groups according to their SpO_2_ levels: low (≤95%), moderate (95–98%), and high (≥98%). To investigate the association between percutaneous oxygen saturation and mortality in patients with severe SAH, logistic regression, and cubic spline models were utilized. The main outcomes of interest were 28- and 90-day mortality rates. Additionally, subgroup analyses were conducted to evaluate these correlations and assess the consistency of interactions.

**Results:**

A cohort of 864 patients diagnosed with non-traumatic SAH was included in this study. The correlation between SpO_2_ and mortality displayed a U-shaped curve when utilizing a finite cubic spline function (non-linearity < 0.001), with the nadir in the probability of in-hospital death at 96%. Mortality at 28 and 90 days showed an inverse correlation with SpO_2_ < 96% (adjusted odds ratio [OR], 0.8; 95% confidence interval [CI], 0.67–0.95, and 0.76; 95% CI, 0.6–0.96). Conversely, there was a positive correlation between percutaneous oxygen saturation (SpO_2_) levels of ≥96% and mortality rates at both 28 and 90 days (adjusted OR, 1.17; 95% CI, 1.02–1.35 and 1.2; 95% CI, 1.05–1.39).

**Conclusion:**

In patients with severe subarachnoid hemorrhage, the association between SpO_2_ and mortality at 28 and 90 days demonstrated a U-shaped pattern. When SpO_2_ levels were between 95 and 98%, both short- and long-term mortality rates were at their lowest. Patients with significant subarachnoid hemorrhage had a lower chance of survival when their SpO_2_ values were either high or low.

## Introduction

Traumatic subarachnoid hemorrhage (SAH), which accounts for 2–7% of all fatalities (1), is a serious and potentially fatal disorder primarily caused by the rupture of cerebral aneurysms. Compared to ischemic stroke and cerebral hemorrhage, non-traumatic subarachnoid hemorrhage is a more severe type of stroke (2), comprising only 5% of all strokes (3). It has an annual incidence of 8 per 100,000 people (4), occurs relatively early in life (3), and is associated with high mortality, morbidity, and poor prognosis when compared to more common strokes (5). Population-based research indicates that the median age of non-traumatic SAH patients is 55 years, and they have a morbidity and mortality rate of approximately 50% (2, 6, 7). One-third of SAH patients pass away before reaching the hospital, and the remaining patients require ICU care (1, 5). Despite adequate therapeutic care in the ICU, patients with severe SAH continue to experience a significant rate of mortality (8). Reports suggest that 30% of SAH patients die within 48 h of admission, 56% die after the first week, and 76% die after the second week (9).

Transcutaneous oxygen saturation measures how much oxygen is bound to hemoglobin in arterial blood and can be used to monitor cardiovascular disease, determine the respiratory–circulatory condition of ICU patients, and determine whether or not the blood oxygen level is normal (10). SpO_2_ has the benefit of being able to continually measure blood oxygen levels, dynamically reflecting the hypoxic or hyperoxic status of ICU patients, as well as being simple to monitor and observe and non-invasive. It also has an advantage over partial pressure of arterial oxygen (PaO_2_) and arterial oxygen saturation (SaO_2_) (11–15).

The detrimental effects of hypoxia are well-established, and SpO_2_ is valuable for monitoring hypoxemia. However, hyperoxia can also have negative consequences (16–22). Increased oxidative stress and inflammation resulting from excessive oxygen supplementation can lead to lung and systemic damage (23). Moreover, studies have demonstrated that elevated levels of oxygen saturation can adversely affect the central nervous system (24). Oxygen therapy lacks a defined standard in many therapeutic situations, and its connection with mortality among individuals with severe subarachnoid hemorrhage remains unknown. While studies have discussed the impact of transcutaneous oxygen saturation on in-hospital mortality in intensive care patients with brain injury (25), patients with severe non-traumatic subarachnoid hemorrhage have not been extensively investigated.

Therefore, this study utilizes data from a large intensive care database to examine the relationship between transcutaneous oxygen saturation and death in patients with critical subarachnoid hemorrhage.

## Materials and methods

### Study population

The Critical Care Medical Information Marketplace MIMIC-IV (v2.2) database, maintained by the MIT Laboratory of Computational Physiology,^1^ was utilized in this study and contains all the necessary data. This database is a comprehensive, open-access, well-documented, and freely available longitudinal single-center database that encompasses inpatient information from cases accepted into the Boston Higher Medical Center in Boston, Massachusetts, United States, between 2008 and 2019. The extraction of unprocessed patient data from the database and its subsequent development for retrospective investigations was validated by the Beth Israel Deaconess Medical Center (Boston, MA, United States) and the Massachusetts Institute of Technology (Cambridge, MA, United States).

It was justified to utilize the MIMIC-IV (V2.2) database for this study for the following reasons: First, all data within the database were carefully de-identified, meaning patient identities were replaced with randomization numbers. This anonymization process is an integral part of MIMIC-IV’s design, which aims to facilitate various research and instructional endeavors while upholding patient privacy and simplifying the conduct of clinical research. Consequently, the requirement for informed consent was waived by the Ethics Committee at Beth Israel Deaconess Medical Center. It should be highlighted that this study followed the guidelines stated in the Declaration of Helsinki and adhered to the principles outlined in the Statement to Strengthen Reporting of Observational Studies in Epidemiology (STROBE). Furthermore, author JL has satisfactorily completed the Collaborative Institutional Training Initiative (CITI) course and obtained a passing score on the “Data or Sample Study Only” exam (ID: 52698592), granting her access to the database for extracting the information required to analyze the correlation between mortality and SpO_2_ in patients with severe subarachnoid hemorrhage. The diagnosis of SAH determined using the International Classification of Diseases (ICD) includes both the ninth revision (ICD-9) and the tenth revision (ICD-10).

There were 257,366 patients in MIMIC-IV overall from 2008 to 2019, of whom 69,619 were admitted to the ICU. Of these, a total of 3,328 patients were selected based on the following recorded ICD-9 code: 430 and ICD-10 codes: I60, I600 ~ I6012, I6000 ~ I6002, I6020 ~ I6022, I6030 ~ I6032, and I6050 ~ I6052, and 3,328 patients with SAH were included. An analysis was conducted that included patients who satisfied the following criteria for selection: (1) it was their first ICU admission; (2) they were over 18 years of age; and (3) SpO_2_ recordings were available within 24 h before admission. Exclusion criteria included those as follows: (1) clear evidence of trauma as the cause; (2) ICU stay of less than 24 h; (3) ICU readmission; (4) missing data exceeding 5%; (5) lack of follow-up information; (6) ICU stay of fewer than 48 h with fewer than 24 recorded SpO_2_ measures; and (7) patients who had not received oxygen therapy. As a result, this study only comprised 864 patients.

### Data acquisition

The MIMIC-IV database was utilized to extract the following variables on the initial day of ICU admission: (1) Demographic variables: age and insurance status. (2) Vital signs: temperature, respiratory rate, heart rate, and oxygen saturation levels were recorded on the first day of ICU admission. (3) Comorbidities: comorbidity index, heart failure, atrial fibrillation, vascular disease, stroke, chronic respiratory disease, liver disease, diabetes mellitus, nephropathy, benign tumors, and metastatic solid tumors. (4) Laboratory tests were performed within the initial 24 h after ICU admission, including white blood cell count, platelet count, serum potassium level, serum sodium level, hemoglobin level, blood glucose level, serum chloride level, urea nitrogen level, creatinine level, anion gap, APTT, and other laboratory markers. If a variable was measured multiple times within the previous 24 h, the mean value was used. (5) Glasgow Coma Scale (GCS), Simplified Acute Physiology Score II (SAPS II), and Acute Physiology Score III (APS III) scores were used to assess the severity of illness upon admission. (6) The duration of ICU stay, overall length of hospital stay, occurrences of death within the ICU, and cases of death during hospitalization. Please note that all the data utilized in this study for analysis and research purposes were exclusively extracted from the MIMIC-IV database.

### Endpoints

Endpoints include short-term mortality (28-day mortality) and long-term mortality (90-day mortality).

### Statistics

Regarding continuous variables, statistical measures such as the mean, standard deviation, or median (interquartile range) were employed. Based on the normality of the distribution, either independent sample *t*-tests or Mann–Whitney U-tests were conducted for statistical comparison. To compare non-normally distributed variables between groups, the Wilcoxon rank sum test was utilized. They were also reported as the median interquartile range (IQR). Categorical variable hypotheses were examined using the chi-squared test (or Fisher’s exact method), presenting the number of cases (%) for each analysis.

Based on 95 and 98%, SpO_2_ was split into three groups: 95, 95–98, and 98%. It was investigated how SpO_2_ levels correlated with ICU or hospital outcomes.

Using univariate and multivariate logistic regression models, the association between SpO_2_ levels and mortality rates at both 28- and 90-day intervals in patients diagnosed with severe SAH was examined. Model 1 referred to an unadjusted model; model 2 referred to a minimum adjusted model, adjusted for demographic factors such as sex, age, and race; and model 3 was a maximum adjusted model: adjusted for sex, age, race, insurance status, heart rate, respiratory rate, temperature, GCS score, atrial fibrillation, congestive heart failure, vascular disease, stroke, chronic lung disease, liver disease, diabetes mellitus, renal disease, benign tumors, white blood cell count, hemoglobin, blood chloride, blood glucose, platelet count, creatinine, anion gap, APTT, and length of hospital stay; and model 4 was a comprehensive and fully adjusted model that considered all variables listed in Table 1, ensuring a thorough examination of their impact on the outcomes.

**Table 1:** Baseline characteristics of selected patients grouped by SpO_2_ level.

Variables	Total (*n* = 864)	SpO₂ <95% (*n* = 62)	SpO₂ (≥95%, <98%) (*n* = 352)	SpO₂ (≥98%) (*n* = 450)	*p* value
^*^Age, year	64.0 ± 17.1	72.0 ± 14.2	65.3 ± 16.2	61.9 ± 17.7	< 0.001
^*^Sex, *n* (%)					< 0.001
Female subjects	466 (53.9)	32 (51.6)	176 (50)	258 (57.3)	
Male subjects	398 (46.1)	30 (48.4)	176 (50)	192 (42.7)	
^*^Race, *n* (%)					
White	551 (63.8)	38 (61.3)	272 (77.3)	241 (53.6)	< 0.001
Asian	28 (3.2)	3 (4.8)	7 (2)	18 (4)	
Black	52 (6.0)	3 (4.8)	17 (4.8)	32 (7.1)	
Other	233 (27.0)	18 (29)	56 (15.9)	159 (35.3)	
^*^Insurance, *n* (%)					0.007
Medicaid	57 (6.6)	2 (3.2)	25 (7.1)	30 (6.7)	
Medicare	317 (36.7)	37 (59.7)	127 (36.1)	153 (34)	
Other	490 (56.7)	23 (37.1)	200 (56.8)	267 (59.3)	
^*^HR, beats/min	79.8 ± 14.2	78.5 ± 14.8	78.1 ± 13.4	81.2 ± 14.6	0.006
^*^SBP, mmHg	124.7 ± 14.3	126.4 ± 20.7	126.0 ± 13.4	123.4 ± 13.9	0.023
^*^DBP, mmHg	63.9 ± 9.6	61.0 ± 9.6	64.8 ± 9.4	63.6 ± 9.7	0.009
^*^MBP, mmHg	80.7 ± 9.4	77.8 ± 10	80.9 ± 8.7	80.9 ± 9.7	0.045
RR, beats/min	18.1 ± 3.2	18.9 ± 4	17.9 ± 3.4	18.1 ± 3	0.092
^*^Temperature, °C	37 ± 0.6	36.9 ± 0.8	36.9 ± 0.5	37 ± 0.6	0.002
^*^SAPSII	33.4 ± 13.1	40.1 ± 18.3	30.1 ± 11.9	35 ± 12.6	< 0.001
^*^APSIII	39.0 (28, 56)	42.5 (29, 73.8)	33 (25.8, 46)	44 (30, 61.8)	< 0.001
^*^GCS	13.9 ± 1.9	13.9 ± 2.3	14.1 ± 1.5	13.8 ± 2.2	0.045
^*^Charlson index	4 (3, 6)	5 (4, 6)	4 (3, 6)	4 (3, 6)	< 0.001
Myocardial infarct	60 (6.9)	4 (6.5)	22 (6.2)	34 (7.6)	0.776
Congestive heart failure	73 (8.4)	8 (12.9)	31 (8.8)	34 (7.6)	0.348
Peripheral vascular disease	77 (8.9)	7 (11.3)	31 (8.8)	39 (8.7)	0.791
^*^Cerebrovascular disease	572 (66.2)	47 (75.8)	218 (61.9)	307 (68.2)	0.044
Chronic pulmonary disease	109 (12.6)	12 (19.4)	43 (12.2)	54 (12)	0.252
Mild liver disease	41 (4.7)	2 (3.2)	17 (4.8)	22 (4.9)	0.934
Diabetes	136 (15.7)	13 (21)	51 (14.5)	72 (16)	0.424
Renal disease	65 (7.5)	7 (11.3)	25 (7.1)	33 (7.3)	0.49
Metastatic solid tumor	15 (1.7)	1 (1.6)	5 (1.4)	9 (2)	0.837
*WBC, 10^9^/L	12.4 (9.3, 15.9)	12.1 (9.0, 15.8)	11.8 (8.7, 14.9)	13.2 (10.1, 16.8)	< 0.001
^*^Hemoglobin, g/dL	11.6 ± 2.1	12 ± 1.7	12 ± 2.1	11.2 ± 2	< 0.001
platelets, 10^9^/L	202.6 ± 82.3	209.6 ± 78.3	207.2 ± 87.0	198.1 ± 78.9	0.233
^*^Glucose, mg/dL	135.2 (116.6, 158.8)	143.8 (123.5, 242.9)	132.2 (114.0, 152.9)	136.2 (118.4, 159)	0.007
^*^Sodium, mmol/L	141.1 ± 4.8	145 ± 6	140.3 ± 4.1	141.8 ± 4.9	< 0.001
Potassium, mmol/L	4.3 ± 0.7	4.2 ± 0.5	4.2 ± 0.7	4.3 ± 0.8	0.546
^*^Chloride, mmol/L	107.1 ± 5.6	105.4 ± 6	105.5 ± 5	108.6 ± 5.7	< 0.001
^*^BUN, mg/dL	17 (13, 22.2)	20.5 (16, 28)	17 (13, 23)	16 (12, 22)	< 0.001
^*^Creatinine, mg/dL	0.9 (0.7, 1.1)	1 (0.8, 1.3)	0.9 (0.7, 1.1)	0.9 (0.7, 1.1)	0.023
^*^Bicarbonate, mmoL/L	24.8 ± 3.3	25.4 ± 4.8	25.3 ± 3	24.3 ± 3.1	< 0.001
Anion gap, mmoL/L	16 ± 3.4	16.2 ± 3	15.8 ± 3.2	16.2 ± 3.6	0.364
^*^INR	1.3 ± 0.9	1.4 ± 0.7	1.4 ± 1.3	1.2 ± 0.4	0.045
PT, s	12.8 (11.9, 14.4)	13.4 (12.2, 14.9)	12.8 (11.9, 14.4)	12.8 (11.9, 14.1)	0.133
APTT, s	28.5 (25.6, 33.5)	28.9 (26.4, 34.2)	28.4 (25.6, 33.0)	28.6 (25.4, 33.8)	0.57
^*^ICU LOS (day)	3 (1, 9)	1 (1, 2)	2 (1, 7)	5 (2, 11)	< 0.001
^*^Hospital LOS (day)	8 (4, 15)	3(1, 9)	7 (4, 12)	10 (5, 19)	< 0.001
^*^Hospital mortality, *n* (%)	166 (19.2)	29 (46.8)	44 (12.5)	93 (20.7)	< 0.001
^*^28-day mortality, *n* (%)	191 (22.1)	25 (40.3)	65 (18.5)	101 (22.4)	< 0.001
^*^90-day mortality, *n* (%)	220 (25.5)	28 (45.2)	73 (20.7)	119 (26.4)	< 0.001

SpO₂, Percutaneous oxygen saturation; SBP, Systolic blood pressure; DBP, Diastolic blood pressure; MBP, Mean blood pressure; RR, Respiratory rate; HR, Heart rate; SAPS II, Simplified acute physiology score II; GCS, Glasgow Coma Score; Chronic pulmonary disease, Chronic obstructive pulmonary disease; WBC, White blood cell; BUN, Blood urea nitrogen; INR, International normalized ratio; PT, Prothrombin time; APTT, Activated partial thromboplastin time; ICU LOS, Intensive care unit length of stay; Hospital LOS, Hospital length of stay.

Additionally, we employed stratified linear regression models, likelihood ratio tests, and curve fitting to establish the correlation between SpO_2_ levels and ICU or in-hospital mortality rates. In the study, we had the following data: age (<60 or ≥ 60 years), GCS score (<8 or ≥ 8), SAPS II score (<30 or ≥ 30), and length of ICU stay (<7 or ≥ 7 days). Statistical analysis was conducted using the R software package (R Foundation, http://www.R-project.org) and Free Statistics software version 1.7. A value of *p* < 0.05 was employed as the threshold for determining statistical significance.

## Results

### Baseline characteristics of the participants

**Figure 1:**
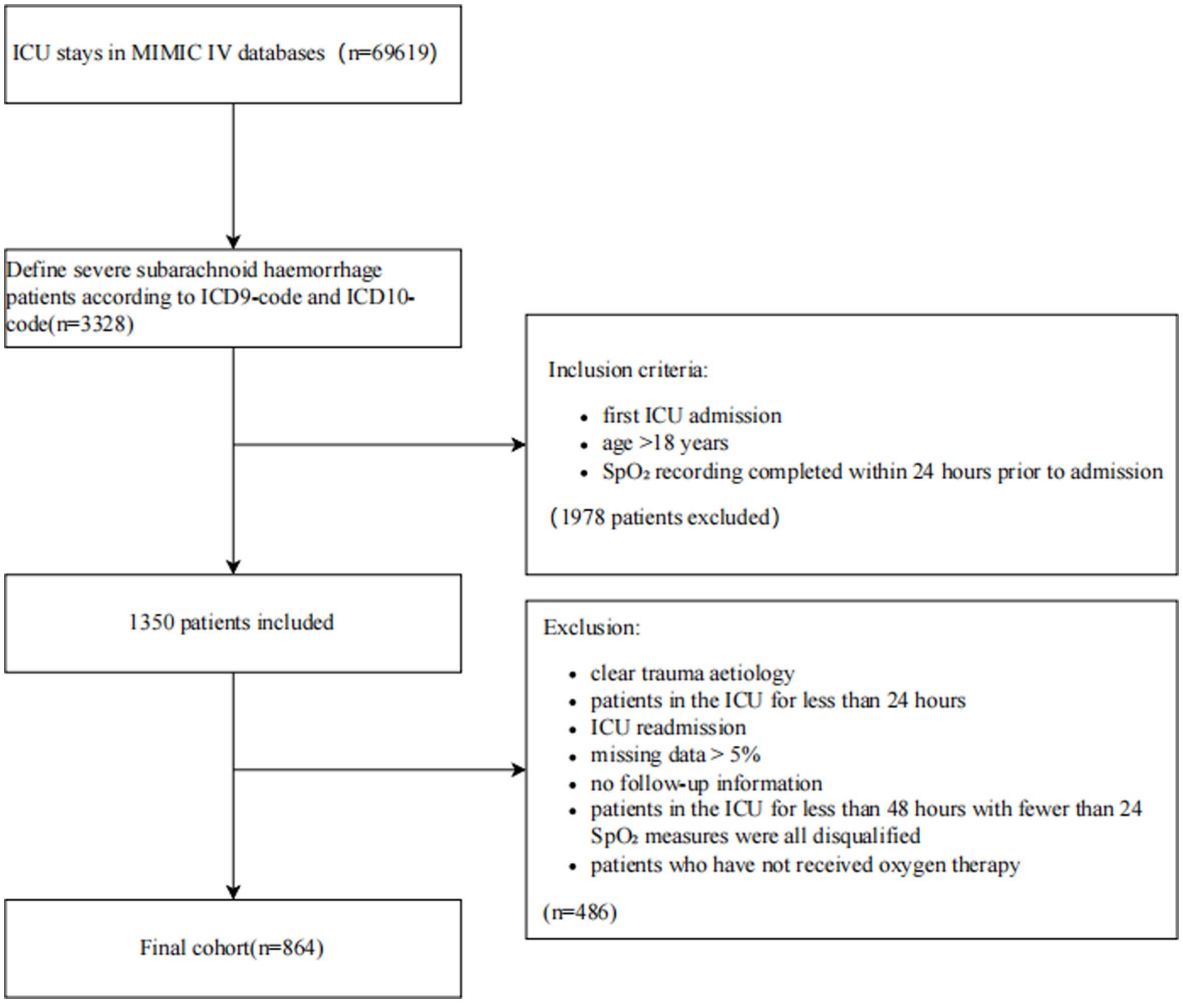
Patient selection flowchart. ICD10, Tenth version of the International Classification of Disease; ICD9, Ninth version of the International Classification of Disease; ICU, Intensive care unit; MIMIC-IV, Medical information mart for intensive care fourth edition; SpO_2_, Pulse oximetry-derived oxygen saturation.

The analysis included 864 consecutive participants (Figure 1). Their SpO_2_ levels were categorized into three groups using thresholds of 95 and 98%. Table 1 provides an overview of the baseline characteristics, revealing that the average age of the participants was 64.0 ± 17.1 years, with men accounting for 46.1% of the study population. Participants with high SpO_2_ (≥98%) were younger and mostly women. Mortality at 28 and 90 days was 22.1 and 25.5%, respectively. The low SpO_2_ (<95%) group had the highest mortality rate, followed by the high SpO_2_ (≥98%) group (*p* < 0.05). In the high SpO_2_ (≥98%) group, several notable differences were observed when compared to other racial backgrounds. These differences included a higher heart rate, lower systolic blood pressure, elevated body temperature, higher APS III score, lower GCS score, increased white blood cell count, lower hemoglobin level, higher chloride ion level, shorter ICU stay, and shorter overall hospital stay (*p* < 0.05). In the low SpO_2_ (<95%) group, participants displayed distinct characteristics, including higher systolic blood pressure, lower diastolic blood pressure, decreased mean arterial pressure, accelerated respiratory rate, higher SAPS II score, elevated BUN level, prolonged ICU stay, and extended overall hospital stay (*p* < 0.05). However, no significant differences were observed among the three groups in terms of complications and relevant parameters (*p* < 0.05).

### The impact of percutaneous oxygen saturation on 28- and 90-day mortality rates

In Table 2, the results of univariate logistic regression analysis demonstrated significant correlations between several factors and the 28- and 90-day mortality rates. These factors included age, insurance status, heart rate, body temperature, respiratory rate, oxygen saturation, comorbidity index, congestive heart failure, chronic respiratory disease, renal disease, metastatic solid tumors, white blood cell count, hemoglobin level, serum sodium, serum chloride, blood urea nitrogen, anion gap, and hospitalization duration (*p* < 0.05).

**Table 2:** Univariate logistic analysis between oxygen saturation and 28- or 90-day mortality.

Variable	28-day mortality		90-day mortality	
	OR (95%CI)	*p* value	OR (95%CI)	*p* value
^*^Age	1.04 (1.03 ~ 1.05)	<0.001	1.04 (1.02 ~ 1.05)	<0.001
Sex: male subjects vs. female subjects	1.21 (0.88 ~ 1.67)	0.249	1.21 (0.89 ~ 1.64)	0.231
Race, *n* (%)				
White	1		1	
Asian	1.55 (0.67 ~ 3.61)	0.309	1.55 (0.68 ~ 3.51)	0.294
Black	0.7 (0.32 ~ 1.54)	0.38	0.78 (0.38 ~ 1.6)	0.495
^*^Other	1.41 (0.98 ~ 2.01)	0.061	1.46 (1.04 ~ 2.06)	0.029
Insurance				
Medicaid	1		1	
^*^Medicare	4.33 (1.8 ~ 10.41)	0.001	4.41 (1.94 ~ 10.04)	<0.001
Other	1.61 (0.67 ~ 3.88)	0.289	1.65 (0.73 ~ 3.76)	0.232
^*^HR, beats/min	1.01 (1 ~ 1.02)	0.096	1.01 (1 ~ 1.02)	0.023
SBP, mmHg	1.01 (1 ~ 1.02)	0.06	1.01 (1 ~ 1.02)	0.168
DBP, mmHg	0.99 (0.97 ~ 1.01)	0.176	0.99 (0.98 ~ 1.01)	0.401
MBP, mmHg	1 (0.98 ~ 1.02)	0.899	1 (0.99 ~ 1.02)	0.761
^*^RR, beats/min	1.16 (1.1 ~ 1.21)	<0.001	1.17 (1.12 ~ 1.23)	<0.001
^*^Temperature, °C	0.87 (0.66 ~ 1.14)	0.308	0.75 (0.58 ~ 0.97)	0.028
^*^SpO₂, mean ± SD	0.91 (0.86 ~ 0.97)	0.004	0.92 (0.86 ~ 0.98)	0.008
^*^SAPSII	1.07 (1.06 ~ 1.09)	<0.001	1.07 (1.06 ~ 1.09)	<0.001
^*^APSIII	1.03 (1.03 ~ 1.04)	<0.001	1.04 (1.03 ~ 1.04)	<0.001
^*^GCS	0.82 (0.76 ~ 0.89)	<0.001	0.82 (0.76 ~ 0.88)	<0.001
^*^Charlson comorbidity index	1.28 (1.2 ~ 1.38)	<0.001	1.29 (1.21 ~ 1.38)	<0.001
^*^Myocardial infarct	1.7 (0.96 ~ 3.01)	0.067	1.77 (1.02 ~ 3.07)	0.041
^*^Congestive heart failure	2.24 (1.35 ~ 3.72)	0.002	2.21 (1.35 ~ 3.62)	0.002
Peripheral vascular disease	0.92 (0.52 ~ 1.63)	0.769	0.96 (0.56 ~ 1.64)	0.868
Cerebrovascular disease	1.15 (0.81 ~ 1.62)	0.43	1.16 (0.84 ~ 1.61)	0.377
^*^Chronic pulmonary disease	1.73 (1.11 ~ 2.69)	0.015	1.61 (1.04 ~ 2.47)	0.031
^*^Mild liver disease	1.89 (0.97 ~ 3.69)	0.061	1.94 (1.02 ~ 3.71)	0.044
Diabetes	1.27 (0.83 ~ 1.94)	0.267	1.44 (0.97 ~ 2.15)	0.074
^*^Renal disease	3.17 (1.89 ~ 5.32)	<0.001	2.94 (1.76 ~ 4.92)	<0.001
^*^Metastatic solid tumor	4.16 (1.49 ~ 11.62)	0.007	4.54 (1.6 ~ 12.89)	0.005
^*^WBC, 10^9^/L	1.02 (1 ~ 1.05)	0.044	1.03 (1 ~ 1.05)	0.018
^*^Hemoglobin, g/dL	0.89 (0.83 ~ 0.97)	0.004	0.89 (0.83 ~ 0.96)	0.002
^*^Platelets, 10^9^/L	1 (0.99 ~ 1)	0.002	1 (0.99 ~ 1)	0.002
Glucose, mg/dL	1 (1 ~ 1)	0.598	1 (1 ~ 1)	0.569
^*^Sodium, mmol/L	1.07 (1.04 ~ 1.11)	<0.001	1.08 (1.04 ~ 1.12)	<0.001
^*^Potassium, mmol/L	1.32 (1.08 ~ 1.62)	0.006	1.32 (1.09 ~ 1.61)	0.005
^*^Chloride, mmol/L	1.04 (1.01 ~ 1.07)	0.004	1.05 (1.02 ~ 1.08)	<0.001
^*^BUN, mg/dL	1.04 (1.03 ~ 1.06)	<0.001	1.04 (1.03 ~ 1.06)	<0.001
^*^Creatinine, mg/dL	1.25 (1.04 ~ 1.5)	0.019	1.25 (1.04 ~ 1.5)	0.02
Bicarbonate, mmoL/L	0.97 (0.93 ~ 1.02)	0.286	0.97 (0.92 ~ 1.02)	0.19
^*^Anion gap, mmoL/L	1.1 (1.05 ~ 1.15)	<0.001	1.1 (1.05 ~ 1.15)	<0.001
INR	1.06 (0.91 ~ 1.24)	0.434	1.1 (0.95 ~ 1.28)	0.218
PT, s	1.01 (0.99 ~ 1.02)	0.431	1.01 (1 ~ 1.03)	0.173
APTT, s	1 (1 ~ 1.01)	0.335	1 (1 ~ 1.01)	0.261
^*^ICU LOS (day)	0.97 (0.94 ~ 0.99)	0.014	0.97 (0.95 ~ 0.99)	0.011
^*^Hospital LOS (day)	0.95 (0.93 ~ 0.97)	<0.001	0.95 (0.93 ~ 0.97)	<0.001

SpO₂, Percutaneous oxygen saturation; SBP, Systolic blood pressure; DBP, Diastolic blood pressure; MBP, Mean blood pressure; RR, Respiratory rate; HR, Heart rate; SAPS II, Simplified acute physiology score II; GCS, Glasgow Coma Score; Chronic pulmonary disease, Chronic obstructive pulmonary disease; WBC, White blood cell; BUN, Blood urea nitrogen; INR, International normalized ratio; PT, Prothrombin time; APTT, Activated partial thromboplastin time; ICU LOS, Intensive care unit length of stay; and Hospital LOS, Hospital length of stay.

In the multivariable logistic model, we observed consistent associations between SpO_2_ and the 28- and 90-day mortality rates across all four models (Table 3). When analyzing SpO_2_ as a continuous variable, we found a negative correlation with mortality rates. Furthermore, when SpO_2_ was categorized, the SpO_2_ (<95%) group exhibited an increased risk of mortality compared to the SpO_2_ (95–98%) group in critically ill SAH patients (adjusted OR, 1.67; 95% CI, 1.08–3.46 for 28-day mortality, and 1.79; 95% CI, 1.02–3.65 for 90-day mortality). In the fully adjusted model, which took into account all covariates listed in Table 1, the SpO_2_ (>98%) group showed an increased risk of mortality compared to the SpO_2_ (95–98%) group in critically ill SAH patients (adjusted OR, 1.14; 95% CI, 1.01–1.36 for 28-day mortality, and 1.36; 95% CI, 1.12–1.47 for 90-day mortality). However, no significant relationship was found between high oxygen saturation (SpO_2_ > 98%) and mortality rates in critically ill SAH patients in the unadjusted model, minimal adjustment model, and maximal adjustment model (which included various demographic and clinical factors).

**Table 3:** Multivariate logistic analysis between percutaneous oxygen saturation and 28- or 90-day mortality.

Variable	Non-adjusted model		Minimally adjusted model		Multiply adjusted model		Fully adjusted model	
	OR (95% CI)	*p*-value	OR (95% CI)	*p*-value	OR (95% CI)	*p*-value	OR (95% CI)	*p*-value
28-day mortality								
^*^SpO₂ (%)	0.91 (0.86 ~ 0.97)	0.004	0.94 (0.88 ~ 1.01)	0.07	0.96 (0.89 ~ 1.04)	0.301	0.91 (0.84 ~ 0.99)	0.033
SpO₂ tertiles								
^*^Low (<95%)	2.98 (1.68 ~ 5.3)	<0.001	2.36 (1.29 ~ 4.28)	0.005	1.77 (1.1 ~ 3.48)	0.06	1.67 (1.08 ~ 3.46)	0.046
Medium (95–98%)	Reference		Reference		Reference		Reference	
High (>98%)	1.28 (0.9 ~ 1.81)	0.168	1.35 (0.93 ~ 1.96)	0.114	1.24 (0.81 ~ 1.92)	0.324	1.14 (1.01 ~ 1.36)	0.502
*p* for trend	0.85 (0.66 ~ 1.09)	0.201	0.93 (0.72 ~ 1.21)	0.602	0.96 (0.7 ~ 1.31)	0.792	0.75 (0.54 ~ 1.05)	0.094
90-day mortality								
^*^SpO₂ (%)	0.92 (0.86 ~ 0.98)	0.008	0.95 (0.89 ~ 1.01)	0.1	0.97 (0.9 ~ 1.05)	0.481	0.92 (0.85 ~ 1)	0.041
SpO₂ tertiles								
^*^Low (<95%)	3.15 (1.79 ~ 5.52)	<0.001	2.5 (1.39 ~ 4.49)	0.002	1.82 (0.93 ~ 3.55)	0.08	1.79 (1.02 ~ 3.65)	0.012
Medium (95–98%)	Reference		Reference		Reference		Reference	
High (>98%)	1.37 (0.99 ~ 1.92)	0.061	1.45 (1.02 ~ 2.07)	0.04	1.38 (0.91 ~ 2.09)	0.129	1.36 (1.12 ~ 1.47)	0.767
*p* for trend	0.89 (0.7 ~ 1.13)	0.33	0.97 (0.75 ~ 1.25)	0.816	1.03 (0.76 ~ 1.39)	0.86	0.79 (0.57 ~ 1.09)	0.146

Unadjusted: Unadjusted for covariates. Minimally adjusted model: Adjusted for age, sex, and race. Multivariable adjusted model: Adjusted for age, sex, race, insurance status, heart rate, respiratory rate, temperature, GCS score, congestive heart failure, atrial fibrillation, vascular disease, stroke, chronic lung disease, liver disease, diabetes, renal disease, benign tumor, white blood cell count, hemoglobin, platelet count, blood glucose, blood chloride, creatinine, anion gap, APTT, and length of hospital stay. Fully adjusted model: Adjusted for all covariates listed in Table 1. SpO₂, Percutaneous oxygen saturation; OR, Odds ratio; CI, Confidence interval.

**Figure 2:**
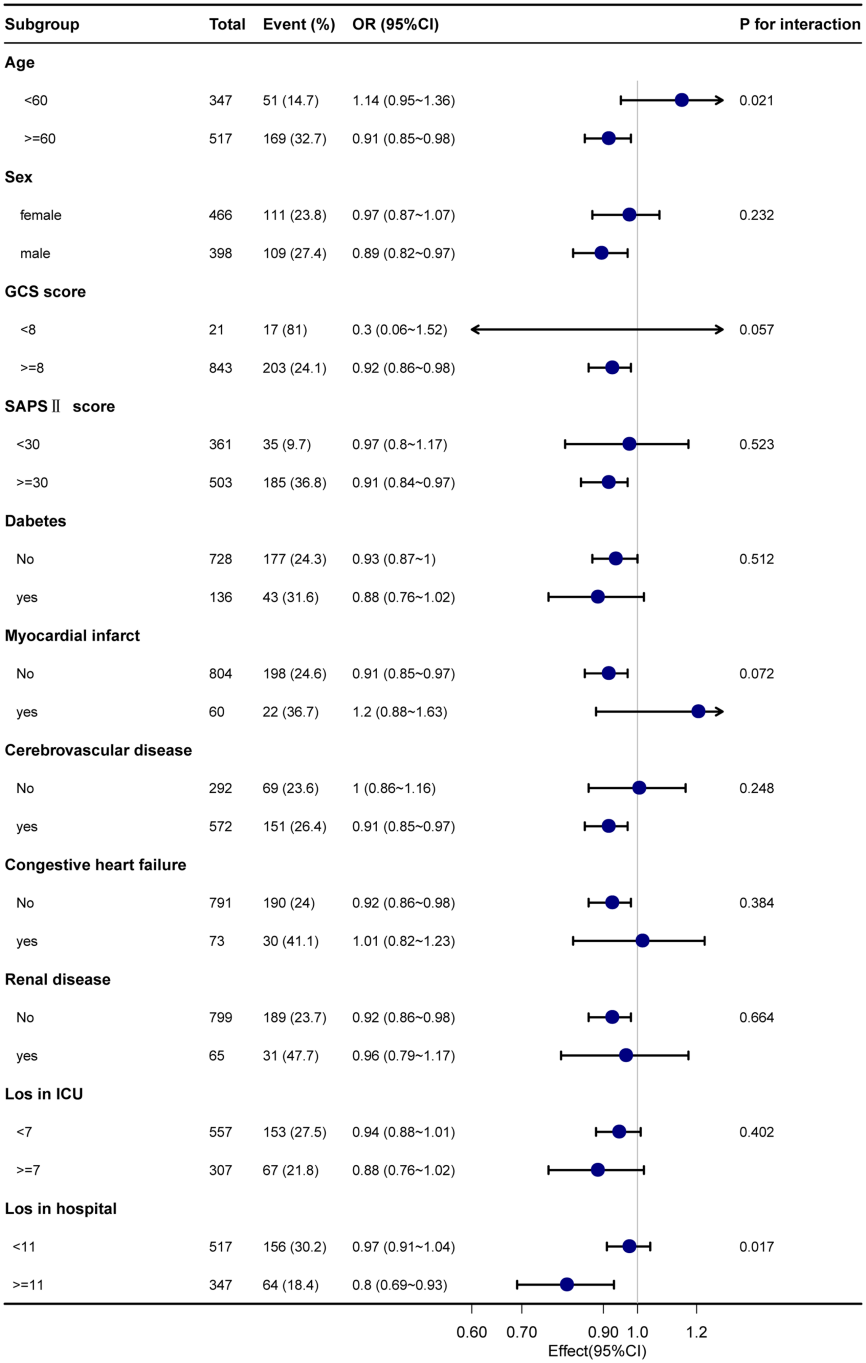
Smoothed curves depicting the relationship between Sp0_2_ levels and the risk of mortality, constructed using a restricted cubic spline model. **(A)** Short-term (28-day) mortality rate; **(B)** Long-term (90-day) mortality rate. The solid black line represents the fitted curve between Sp0_2_ and mortality, while the gray shaded area indicates the corresponding 95% confidence interval. The model was adjusted for multiple covariates, including age, sex, race, insurance status, heart rate, respiratory rate, temperature, GCS score, congestive heart failure, atrial fibrillation, vascular disease, stroke, chronic lung disease, liver disease, diabetes, renal disease, benign tumor, white blood cell count, hemoglobin, platelet count, blood glucose, blood chloride, creatinine, anion gap, activated partial thromboplastin time (APTT), and length of hospital stay. The blue shadow in the figure represents the relative density curve of overall cases, while the red shadow represents the relative density curve of positive outcome cases. It has been explained in the caption.

Figure 2 illustrates the correlation between average SpO_2_ and both 28- and 90-day mortality rates. It is noteworthy that both hypoxemia and hyperoxemia are associated with an increased risk of mortality. As a result, we have identified a specific range of SpO_2_ values that carries a lower risk of mortality. To account for various factors such as age, sex, race, GCS score, SAPS II score, APS III score, and length of hospital stay, we employed restricted cubic splines to analyze the relationship between SpO_2_ and mortality rates in SAH patients. Our findings revealed a non-linear, U-shaped pattern in the relationship between SpO_2_ and in-hospital mortality (*p* < 0.001). The nadir of the mortality risk was observed at 96% SpO_2_. As the saturation level increased beyond this point, a less pronounced U-shaped curve was observed. It is worth mentioning that, even without precise information on corresponding PaO_2_ levels, we can infer that higher oxygen levels are relatively less harmful than hypoxia.

Using a two-part logistic regression model, we identified a critical threshold of 96% for SpO_2_ (as shown in Table 4). When SpO_2_ levels were below 96%, we observed a negative association with both 28-day and 90-day mortality rates (adjusted OR, 0.8; 95% CI, 0.67–0.95 and 0.76; 95% CI, 0.6–0.96). Conversely, when SpO_2_ levels were equal to or higher than 96%, we found a positive association with 28-day and 90-day mortality rates (adjusted OR, 1.17; 95% CI, 1.02–1.35 and 1.2; 95% CI, 1.05–1.39). These results highlight a clear inflection point at the SpO_2_ threshold of 96%, indicating a significant change in mortality risk as SpO_2_ levels fall below or rise above this critical value. It is important to note that these associations indicate a change in mortality risk as SpO_2_ levels fall below or rise above the 96% threshold.

**Table 4:** Threshold analysis of SpO_2_ on 28- and 90-day mortality in SAH patients in the ICU using a two-segment regression model.

Variable	*n*.total	Non-adjusted model		Multiply adjusted model	
OR (95% CI)		*p* value	OR (95% CI)	*p* value
28-day mortality					
SpO₂ (<96%)	145	0.77 (0.66 ~ 0.91)	0.002	0.8 (0.67 ~ 0.95)	0.012
SpO₂ (≥96%)	719	1.12 (0.97 ~ 1.28)	0.112	1.17 (1.02 ~ 1.35)	0.028
90-day mortality					
SpO₂ (<96%)	145	0.69 (0.56 ~ 0.85)	<0.001	0.76 (0.6 ~ 0.96)	0.02
SpO₂ (≥96%)	719	1.16 (1.02 ~ 1.32)	0.027	1.2 (1.05 ~ 1.39)	0.01

Unadjusted: Unadjusted for covariates. Multivariable adjusted model: Adjusted for age, sex, race, insurance status, heart rate, respiratory rate, temperature, GCS score, congestive heart failure, atrial fibrillation, vascular disease, stroke, chronic lung disease, liver disease, diabetes, renal disease, benign tumor, white blood cell count, hemoglobin, platelet count, blood glucose, blood chloride, creatinine, anion gap, APTT, and length of hospital stay. SpO₂, Percutaneous oxygen saturation; OR, Odds ratio; and CI, Confidence interval.

### Sensitivity analysis

**Figure 3:**
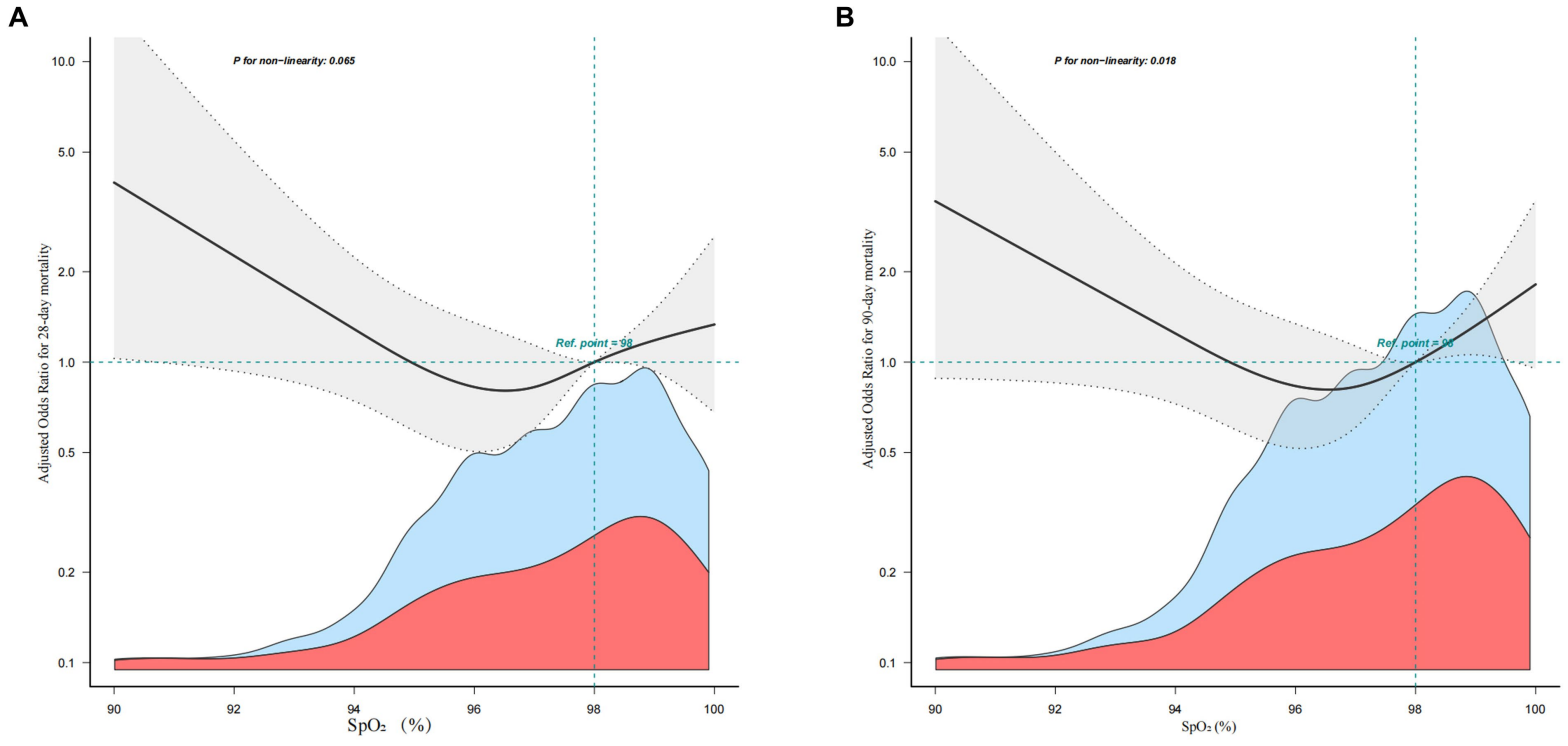
The relationship between Sp0_2_ and 28-day mortality in subgroup analysis.

**Figure 4:**
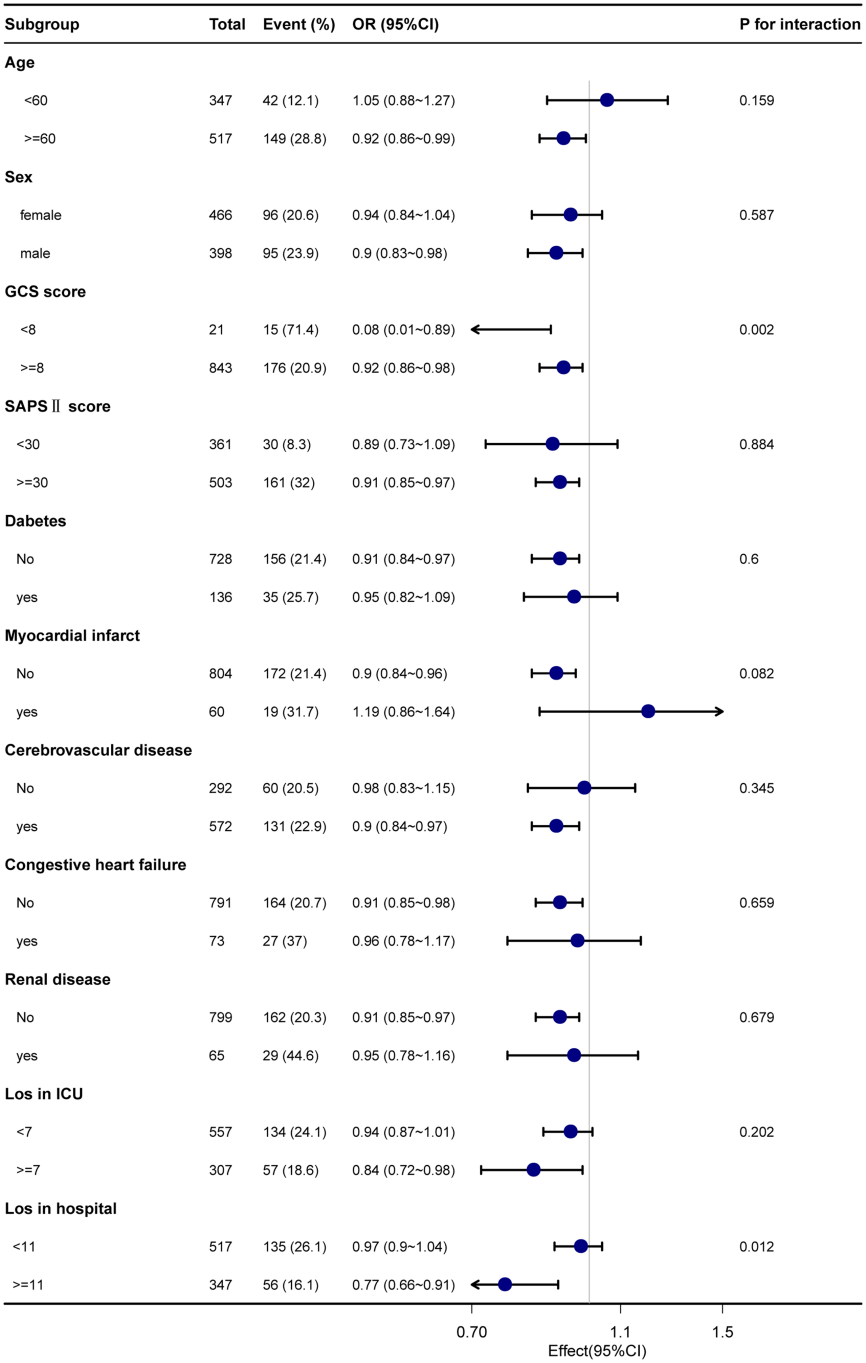
The relationship between Sp0_2_ and 90-day mortality in subgroup analysis.

Subgroup analyses were performed considering confounding factors such as age, sex, GCS score, SAPS II score, comorbidities (diabetes, myocardial infarction, cerebrovascular disease, congestive heart failure, and renal disease), ICU length of stay, and total length of hospital stay (refer to Figures 3, 4). The results remained robust after these analyses. An interaction was observed between the GCS score and the total length of hospital stay for the 28-day mortality rate. Similarly, an interaction was found among age, GCS score, and total length of hospital stay for the 90-day mortality rate. However, no significant interactions were detected among the other variables (*p* > 0.05). These subgroup analyses strengthen our findings by considering potential confounders and demonstrating the consistency of the results across various subgroups, supporting the reliability of our findings.

## Discussion

This study undertook an investigation into the relationship between SpO_2_ levels and mortality rates among individuals afflicted with subarachnoid hemorrhage (SAH). Specifically, we investigated the association between average SpO_2_ within 24 h of admission and both short-term (28-day) and long-term (90-day) mortality rates. Our findings unveiled an independent correlation between SpO_2_ levels and the risk of mortality in SAH patients. Regardless of the approach taken, whether analyzing SpO_2_ as a continuous or categorical variable, we observed a significant association between SpO_2_ levels and both short-term and long-term mortality rates. Additionally, our analysis using restricted cubic splines indicated a distinctive U-shaped curve in the relationship between SpO_2_ and mortality rates. This U-shaped curve suggested that there was a nadir of mortality risk at approximately 96% SpO_2_, indicating that mortality rates were the lowest within this range. Notably, the significance of these associations persisted even after meticulous adjustment for potential confounding factors. It is noteworthy that maintaining SpO_2_ levels within the range of 95–98% appeared to be beneficial for patient outcomes, as this range was associated with the lowest short-term and long-term mortality rates. Deviations from this optimal range, either above or below, had negative effects on patient survival. Overall, our study emphasizes the independent correlation between SpO_2_ levels assessed during the initial 24 h of admission and subsequent mortality rates among individuals diagnosed with SAH. These findings underscore the importance of monitoring and optimizing SpO_2_ levels to improve patient outcomes in this specific population. The correlation between SpO_2_ and PaO_2_ is deemed reliable when SpO_2_ levels are within the range of 95–98%. This range effectively reduces the likelihood of underestimating hypoxemia or hyperoxemia occurrences (26). Furthermore, the therapeutic benefits of SpO_2_ lie in its capacity to facilitate adjustments in inspired oxygen and ventilator settings according to fluctuations in SpO_2_ readings. This obviates the necessity for intermittent arterial blood gas measurements, providing a more streamlined approach to patient management (27).

It was believed that higher blood oxygen saturation (SpO_2_) resulted in better patient survival rates. However, our research findings have challenged this assumption. Our findings revealed that the group exhibiting elevated SpO_2_ levels had increased rates of both short-term and long-term mortality compared to the group with moderate SpO_2_. Similar studies have also reported similar results. For instance, Davis et al. (28) conducted a retrospective study involving 3,420 TBI patients and found a correlation between extremely high oxygen levels [PaO_2_ (487 mmHg)] upon admission and worse outcomes. In this study, the optimal PaO_2_ range was found to be between 110 and 487 mmHg (28). Another study highlighted the detrimental effects of high oxygen saturation in hospitalized patients, which encompassed prolonged hospitalization and elevated mortality rates within the healthcare setting (29). Furthermore, the lowest mortality rate among TBI patients was observed when SpO_2_ ranged from 94 to 98% (30), which aligns with our research findings. Currently, most research and guidelines focus on TBI patients, and there is a lack of specific research on SAH. It is worth noting that these sources emphasize the identification and correction of hypoxia in the ICU but do not address the potential harm of high oxygen levels (31). Previously, it was believed that higher oxygen saturation would lead to better patient survival rates. However, research suggests that patients with oxygen saturation levels between 98 and 100% have lower survival rates compared to those within the range of 96 and 98%.

Elevated blood oxygen levels can induce vasoconstriction in critical arterial territories, including the brain and coronary arteries, thereby aggravating post-SAH cerebral vasospasm (32). From a molecular biology standpoint, excessively elevated blood oxygen levels can lead to the generation of reactive oxygen species (ROS) as byproducts of aerobic metabolism. These ROS have the potential to cause harm to essential biological elements, such as proteins, lipids, and deoxyribonucleic acid (DNA) (33). Prolonged exposure to excessive hyperoxia invariably leads to tissue damage, causing subsequent neuronal injury, and impairment (34). Furthermore, studies have shown that excessive oxygen supplementation can increase oxidative stress and inflammation, potentially causing negative effects on both the lungs and the overall body (23).

Hypoxia is a major contributing factor to adverse outcomes in stroke, and numerous studies have recommended active oxygen therapy (35, 36). Our research has produced comparable findings, indicating significantly elevated mortality rates at both 28- and 90-day follow-ups in the low SpO_2_ group when compared to the group with moderate SpO_2_ levels. It is well known that hypoxia can lead to various problems in different systems of the human body. Many studies have indicated that hypoxia may disrupt the blood–brain barrier, cause irreversible neurological damage, and result in significant impairment (37). The vascular system in SAH is often compromised, leading to inadequate cerebral perfusion, ischemia, hypoxia, and other complications, with hypoxia exacerbating brain tissue damage. From a molecular biology standpoint, hypoxia-induced aberrant metabolism in the brain tissue sets off a cascade of events, including the development of cerebral edema and disruption of the blood–brain barrier. This creates a cyclical process that perpetuates the detrimental effects (38). Furthermore, hypoxia also causes harm to other systems in the body. Hyperoxia primarily affects the cardiovascular system by manifesting as an elevation in heart rate, increased contractility of the myocardium, impaired diastolic function, and the initial onset of arrhythmias (31). Hypoxia leads to rapid and deep breathing, and over time, respiratory muscles may become fatigued (39). In conclusion, hypoxia causes multi-system damage in SAH patients, impeding their recovery and accelerating mortality.

However, this study has several noteworthy limitations. First, within the MIMIC-IV database, we lacked access to important covariates such as delayed cerebral ischemia, hydrocephalus, intracranial hypertension, and other potential confounding factors. Additionally, there may be residual confounding variables present, as is common in retrospective analyses. Despite employing interpolation techniques, the limited availability of extensive data made it challenging to completely mitigate biases. Furthermore, this study was conducted at a single center, thereby limiting its generalizability to patients from diverse geographic regions. Moreover, the observed relationship between SpO_2_ and overall mortality rates established an association rather than a direct causal relationship. Therefore, it is crucial to conduct additional prospective studies to validate the findings and conclusions derived from this research.

## Conclusion

In non-traumatic SAH patients, we have observed a U-shaped correlation between the initial SpO_2_ levels upon admission and the mortality rates at 28 and 90 days. Additionally, the lowest risks of short-term and long-term mortality were found when the average SPO₂ within the first 24 h of admission was at 96%. Elevated or reduced average SpO_2_ levels during the initial 24 h after admission were both linked to higher mortality rates. Nevertheless, it is worth emphasizing that additional prospective studies are required to substantiate these results.

## Data availability statement

The data analyzed in this study was obtained from the Medical Information Mart for Intensive Care IV (MIMIC-IV) database. The following licenses/restrictions apply: To access the files, users must be credentialed users, complete the required training (CITI Data or Specimens Only Research), and sign the data use agreement for the project. Requests to access these datasets should be directed to PhysioNet, https://physionet.org/, DOI: 10.13026/6mm1-ek67.

## Ethics statement

Ethical review and approval were not required for the study on human participants in accordance with local legislation and institutional requirements. Written informed consent from the patients/participants or patients/participants’ legal guardian/next of kin was not required to participate in this study in accordance with the national legislation and the institutional requirements.

## Author contributions

JuL: Writing – original draft. ZZ: Writing – review & editing. JiL: Conceptualization, Writing – review & editing. QZ: Data curation, Writing – review & editing. YW: Investigation, Writing – review & editing. JZ: Validation, Writing – review & editing.

## Footnotes

^1^ https://physionet.org/content/mimiciv/
